# Total and Nonheme Dietary Iron Intake Is Associated with Metabolic Syndrome and Its Components in Chinese Men and Women

**DOI:** 10.3390/nu10111663

**Published:** 2018-11-04

**Authors:** Zhenni Zhu, Fan Wu, Ye Lu, Chunfeng Wu, Zhengyuan Wang, Jiajie Zang, Changyi Guo, Xiaodong Jia, Jiahui Yao, Hui Peng, Yuna He, Jing Sun, Jian Huang, Gangqiang Ding

**Affiliations:** 1 National Institute for Nutrition and Health, Chinese Center for Disease Control and Prevention, 27 Nanwei Road, Xicheng District, Beijing 100050, China; zhuzhenni@scdc.sh.cn (Z.Z.); heyn@ninh.chinacdc.cn (Y.H.); sunjing@ninh.chinacdc.cn (J.S.); huangjian@ninh.chinacdc.cn (J.H.); 2 Division of Health Risk Factors Monitoring and Control, Shanghai Municipal Center for Disease Control and Prevention, 1380 West Zhongshan Road, Shanghai 20036, China; wufan@scdc.sh.cn (F.W.); wangzhengyuan@scdc.sh.cn (Z.W.); zangjiajie@scdc.sh.cn (J.Z.); guochangyi@scdc.sh.cn (C.G.); jiaxiaodong@scdc.sh.cn (X.J.); 3 Division of Non-Communicable Diseases Prevention and Control, Shanghai Municipal Center for Disease Control and Prevention, 1380 West Zhongshan Road, Shanghai 20036, China; luye@scdc.sh.cn; 4 Department of Profession Management, Shanghai Municipal Center for Disease Control and Prevention, 1380 West Zhongshan Road, Shanghai 20036, China; wuchunfeng@scdc.sh.cn; 5 Department of Public Health, Shanghai Xuhui District Center for Disease Control and Prevention, 50 Yongchuan Road, Shanghai 200237, China; jeffkokeyjh@126.com; 6 Department of Public Health, Shanghai Jiading District Center for Disease Control and Prevention, 264 Tacheng Road, Shanghai 201800, China; jzhjymzl@126.com

**Keywords:** dietary iron intake, heme iron, nonheme iron, metabolic syndrome, population-based study

## Abstract

The causal relationship between serum ferritin and metabolic syndrome (MetS) remains inconclusive. Dietary iron intake increases serum ferritin. The objective of this study was to evaluate associations of total, heme, and nonheme dietary iron intake with MetS and its components in men and women in metropolitan China. Data from 3099 participants in the Shanghai Diet and Health Survey (SDHS) obtained during 2012–2013 were included in this analysis. Dietary intake was assessed by 24-h diet records from 3 consecutive days. Multivariate generalized linear mixed models were used to evaluate the associations of dietary iron intake with MetS and its components. After adjustment for potential confounders as age, sex, income, physical exercise, smoking status, alcohol use, and energy intake, a positive trend was observed across quartiles of total iron intake and risk of MetS (*p* for trend = 0.022). Compared with the lowest quartile of total iron intake (<12.72 mg/day), the highest quartile (≥21.88 mg/day) had an odds ratio (95% confidence interval), OR (95% CI), of 1.59 (1.15,2.20). In addition, the highest quartile of nonheme iron intake (≥20.10 mg/day) had a 1.44-fold higher risk of MetS compared with the lowest quartile (<11.62 mg/day), and higher risks of MetS components were associated with the third quartiles of total and nonheme iron intake. There was no association between heme iron intake and risk of MetS (*p* for trend = 0.895). Associations for total and nonheme iron intake with MetS risk were found in men but not in women. Total and nonheme dietary iron intake was found to be positively associated with MetS and its components in the adult population in metropolitan China. This research also revealed a gender difference in the association between dietary iron intake and MetS.

## 1. Introduction

China has experienced a cardiovascular disease epidemic in recent decades [1]. Metabolic syndrome (MetS) refers to a constellation of interrelated risk factors that increase the development of both cardiovascular disease and type 2 diabetes mellitus [2]. The current nationwide prevalence of MetS among Chinese adults is 24.2%, which is an astonishing increase compared with the value of 9.8% calculated one decade ago based on the same diagnostic criteria [3,4].

A few studies have demonstrated an association between serum ferritin and MetS [5,6,7,8], but whether MetS is causally related to higher levels of iron metabolic markers or whether higher levels of iron metabolic markers lead to MetS remains inconclusive. Iron overload is characterized physiologically by an increase in the serum ferritin levels [9], and some studies have shown that meat or heme iron intake is related to the ferritin levels in serum [10,11]. However, high levels of serum ferritin can also be produced as a consequence of metabolic disorders, regardless of the iron overload status [12].

Few studies have investigated the relationship between dietary iron intake and MetS, which could potentially address the causative nature of the association between iron metabolic markers and MetS. Iron is present in foods in a heme or nonheme form. Heme iron is in most animal foods, and the rest of the iron in animal or plant food is nonheme iron [13]. Heme iron is more efficiently absorbed than nonheme iron as approximately 25% heme iron and 5% nonheme iron from diet absorbed by body [14]. The Chinese diet is known to be plant-based, which implies a low bioavailability of dietary iron. The primary objective of this study was to evaluate the associations of dietary iron intake, in terms of total, heme and nonheme iron intake, with MetS and its components in the adult population in metropolitan China. Considering the different physiologic mechanisms underlying body iron loss among men and women, it was intended to also analyze these associations stratified by gender. To the best of our knowledge, this population-based study constitutes the first investigation of the relationship between dietary iron intake and MetS in a Chinese population.

## 2. Materials and Methods

### 2.1. Study Population

The data used in this study were obtained from a cross-sectional investigation, the first wave of the Shanghai Diet and Health Survey (SDHS), which was conducted in Shanghai, one of the most developed cities in China, during the period 2012–2013. The SDHS was designed to examine the associations of food consumption, energy and nutrient intake, and behavioral factors with nutrition-related health outcomes among local residents. The investigation enrolled 4504 community-dwelling men and women aged at least 18 years from 54 randomly stratified-sampled communities. The participants were initially selected to constitute a random representative sample of the local adult population (*n* = 1725), and their family members aged at least 18 years were then recruited (approximately 2.6 persons per family). Those who had lived in the area for less than 6 months in total during the last year of the survey were excluded. The Shanghai Municipal Center for Disease Control and Prevention was responsible for the implementation of the SDHS.

Participants with missing anthropometric measurements (*n* = 251) or lack of blood pressure assessed (*n* = 54) or whose blood samples were not collected or tested (*n* = 817) for indicators of MetS diagnosis, as well as those who reported an energy intake less than 300 kcal/d or greater than 3500 kcal/d for the average energy intake (mean ± SD) was 1766 ± 880 kcal in the study population and the mean ± 2SD interval was finally used for the acceptable range (*n* = 51), were excluded. Participants with missing dietary survey information (*n* = 38) or other pertinent covariates (*n* = 194) were also excluded. The data from 3099 participants were ultimately included in the present analysis.

The SDHS was approved by the Shanghai Municipal Center for Disease Control and Prevention’s Institutional Review Board on 31 January 2012. Informed consent was obtained from each participant before the survey. The study complied with the code of ethics of the World Medical Association (Declaration of Helsinki).

### 2.2. Dietary Assessment

The dietary survey included a 24-h dietary record for 3 consecutive days (including 2 weekdays and 1 weekend day) in the consideration of different dietary behavior between working days and rest, as well as the feasibility of investigation. Household condiments mainly containing fat or sodium, including cooking oil, salt, soy sauce, chili sauce, etc., were weighed before and after the 3 survey days. The interviewers were public health doctors from 54 local community health centers who received a standard training course on the recording of dietary information. Each participant was orally instructed to record their daily food intake both at home and out of home on draft paper at the beginning and interviewed face-to-face by interviewers in the consecutive survey days at home. At each survey day, the interviewers collected and checked though the draft paper, and afterward, revised the food weight and transcribed the draft dietary information into a structured form. Furthermore, the participants were instructed not to change their typical diet or physical activity during the survey period. The diet records in the structured form were reviewed by nutrition specialists from local centers of disease control and prevention. No disastrous events such as rain or snow disasters that would have affected the normal food supply occurred during the survey period.

Daily food consumption was calculated from the 3-day, 24-h diet record. The 3-day consumption of condiments based on the calculated weight difference was divided into individual intake according to the eating times in the home and the individuals’ energy intake (only from food) proportion among family members. That consumption of condiments from meals out of home was simulated according to the previously calculated condiments’ densities in food consumed at home. The intake of dietary energy, macronutrients and iron was estimated according to daily food and condiment consumption using the Chinese food composition database [15,16]. Dietary supplements and medications were excluded from nutrient intake. Heme iron was estimated as 40% of the total iron from animal foods, including red meat, poultry, fish, and animal organs, and nonheme iron was calculated as the remaining portion of the total iron from all foods [14,17].

### 2.3. Potential Confounders

Information on each participant’s age, sex, education, income, smoking status, alcohol use, physical activity level, and intentional physical exercise was recorded using an interviewer-administered questionnaire at each participant’s home. The educational level of the participants was reported as years of education. The yearly income was calculated by dividing the total family yearly income by the number of family members. The physical activity level was recorded as sedentary, moderate, or vigorous according to professional and nonprofessional activities. Intentional physical exercise was defined as physical exercise performed for the purpose of health maintenance or fitness. The smoking status was categorized as never smoked, former smoker, or current smoker. With respect to alcohol use, the respondents were classified as follows according to their alcohol consumption during the seven days prior to the interview: lifetime abstainers, the respondent did not consume an alcoholic beverage; nonheavy drinkers (social drinkers), the respondent consumed 5+ standard drinks once (1 day) in the 1-week period; infrequent heavy drinkers (binge drinkers), the respondent consumed 5+ standard drinks on 2–3 days in the 1-week period; or frequent heavy drinkers, the respondent consumed 5+ standard drinks on at least 4 days in the 1-week period.

### 2.4. Anthropometric and Laboratory Measurements

All anthropometric measurements were conducted in the community health centers located in each participant’s community. The waist circumference was measured using a Graham-Field 1340-2 tape measure. The blood pressure was measured three times after a quiet rest for 5 min using an Omron HEM-7071 electronic sphygmomanometer (Omron Healthcare, Kyoto, Japan).

Each participant was asked to fast for more than 10 h, and their blood was then collected and analyzed at the laboratory of the Shanghai Municipal Centers for Disease Control and Prevention. The serum concentrations of glucose, triglycerides, and high-density lipoprotein-cholesterol (HDL-C) were measured using a HITACHI 7080 Automatic Biochemical Analyzer with reagents from Wako Pure Chemical Industries, Ltd. (Tokyo, Japan).

### 2.5. Definition of Metabolic Syndrome

MetS was identified based on the criteria in the US National Cholesterol Education Program Adult Treatment Panel III (NCEP-ATP III) for Asian populations [2], which states that at least three of the following metabolic abnormalities should be present: (1) elevated waist circumference (WC ≥ 90 cm for men and WC ≥ 80 cm for women); (2) elevated triglycerides (triglycerides ≥ 150 mg/dL) or on drug treatment for elevated triglycerides; (3) reduced HDL-C (HDL-C < 40 mg/dL for men and < 50 mg/dL for women) or on drug treatment for reduced HDL-C; (4) elevated blood pressure (systolic blood pressure ≥ 130 mmHg and/or diastolic blood pressure ≥ 85 mmHg) or on antihypertensive drug treatment with a history of hypertension; and (5) elevated fasting glucose (100 mg/dL) or on drug treatment for elevated glucose.

### 2.6. Statistical Analyses

Statistical analyses were conducted using SAS statistical software (v. 9.2; SAS Institute, Cary, NC, USA). Given that the occurrence of MetS might aggregate in families due to a similar genetic background, which would potentially cause aggregation bias, multilevel models were introduced in the analysis. Multivariate generalized linear mixed models for binary data with a logit link function were applied to determine the odds ratios (ORs) and 95% confidence intervals (CIs) of the occurrence of MetS and its five components as the dependent variables based on four tertiles of total, heme, and nonheme dietary iron intake as the independent fixed-effect variables and family aggregation as the random-effect variable. No covariate was included in the crude model. Potential confounders, including age, sex, income, physical activity level, intentional physical exercise, dietary energy intake, smoking status, and alcohol use, were introduced as covariates in the adjusted models. To examine the linear trend between dietary iron intake and the occurrence of MetS, the medians in each quartile of dietary iron intake were used as the independent fixed-effect variables, and the occurrences of MetS and its five components were used as the dependent variables. A two-sided *p* value < 0.05 was considered to indicate statistical significance.

## 3. Results

### 3.1. Characteristics of the Participants

The analysis sample included 3099 participants, consisting of 1430 male adults and 1669 female adults. Among the participants, the total, heme, and nonheme dietary iron intake was 19.7 mg/day, 1.6 mg/day, and 18.1 mg/day, respectively, and the prevalence of MetS was 23.9%. The characteristics of the participants are shown in Table 1.

### 3.2. Dietary Sources of Iron Intake

**Figure 1:**
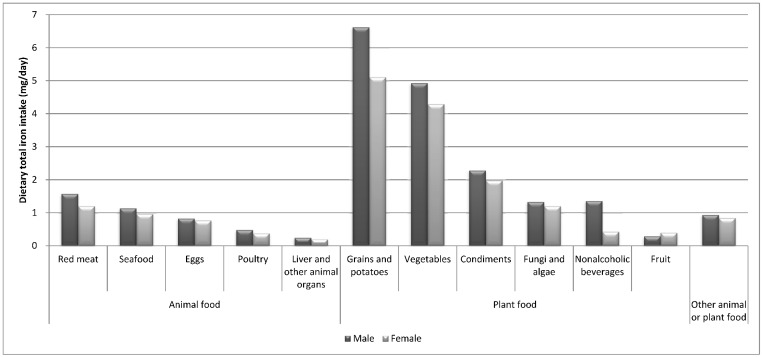
Dietary sources of total iron intake for the different genders in the SDHS 2012–2013.

In present study population, the main dietary sources of total iron intake were plant-based foods. Specifically, the top three dietary sources of total iron intake were grains/potatoes, vegetables, and condiments, and this finding was obtained for both males (6.62 mg/day, 4.92 mg/day, and 2.28 mg/day, respectively) and females (5.10 mg/day, 4.29 mg/day, and 1.97 mg/day, respectively). Red meat was ranked first among the animal food sources of total dietary iron intake consumed by both genders (Figure 1).

### 3.3. Quartiles of Dietary Iron Intake and MetS Risk

After adjusting for age, sex, income, physical activity level, intentional physical exercise, dietary energy intake, smoking status, and alcohol use, a significant positive trend was found across quartiles of total dietary iron intake and risk of MetS (*p* for trend = 0.022). Compared with the lowest quartile of dietary total iron intake (<12.72 mg/day), the highest quartile (≥21.88 mg/day) had an odds ratio (95% confidence interval), OR (95% CI), of 1.59 (1.15,2.20). However, after adjusting for the same confounders, there was no association between heme dietary iron intake and risk of MetS (*p* for trend = 0.895), and compared with the lowest quartile of heme iron intake, the second to fourth tertiles did not show significant differences in ORs. Although the quartiles of nonheme iron intake were not in line with the risk of MetS (*p* for trend = 0.065), the highest quartile of nonheme iron intake (≥20.10 mg/day) had a 1.44-fold higher risk of MetS compared with the lowest quartile (<11.62 mg/day). After adjustment, higher risks for the five components of MetS were observed in the third and fourth quartiles of total and nonheme dietary iron intake. However, no differences in the risks for the MetS components were found between the second to fourth quartiles of heme iron intake and the reference quartile (Table 2, Table 3 and Table 4).

After stratifying by gender, the linear trend between the quartiles of total dietary iron intake and risk of MetS was still significant in male participants (*p* for trend = 0.016) but not in female participants (*p* for trend = 0.322). In men, a higher risk (OR = 2.11, 95% CI: 1.29–3.45) of MetS was observed in the fourth quartile of total dietary iron intake (≥23.68 mg/day) compared with the first quartile (<14.12 mg/day), whereas in women, the risks of MetS were not significantly different between the lowest quartile of total iron intake and the other quartiles. A positive association was found between nonheme iron intake and MetS risk in men (*p* for trend = 0.025) but not in women (*p* for trend = 0.150). Finally, heme iron intake was not associated with MetS in either men or women (Men, Table 5, Table 6 and Table 7; Women, Table 8, Table 9 and Table 10).

After stratifying by age group, a higher risk of MetS was observed in the third and fourth quartile of total dietary iron intake among the age group of above 60 (Table A1).

## 4. Discussion

The current findings were consistent with two previous studies indicating that the consumption of a certain type of dietary iron is related to the risk of developing MetS or its components [18,19]. Compared with the total dietary iron intake of the Western population, the average intake of Chinese adults is reportedly much higher, which is consistent with the iron intake determined in this study [18,20,21,22]. Free iron has strong pro-oxidant properties and generates reactive oxygen species by participating in Fenton chemistry, which results in the induction of oxidative damage and apoptosis [23]. Although iron plays an indispensable role in some physiological processes, excess iron can cause tissue damage or subclinical inflammation [24]. These side effects of iron are balanced out through binding to ferritin, an iron storage protein [13]. Given that several population-based human observational studies have reported a positive association between dietary iron intake and serum ferritin [10,11,21,25,26], the elevated concentration of serum ferritin is regarded as an indicator of iron overload. This research potentially supported the hypothesis that excessive iron in the body might play a causal role in the development of MetS. Nevertheless, MetS has been found to increase the inflammatory status and thereby induces changes in iron homeostasis; thus, the influence of MetS on iron metabolism cannot be ruled out [27,28]. These two mechanisms might mutually correspond to a continuous circle between iron metabolism and MetS.

In present study population, we observed that nonheme iron intake but not heme iron intake was associated with MetS. This finding was inconsistent with studies on Western populations, which concluded that heme iron intake is correlated with MetS [18,20,29]. Dietary iron is present in two forms: heme iron (animal food) and nonheme iron (plant and animal food) [13]. The Chinese diet is well known to be a predominantly plant-based diet. The major food sources of dietary iron in the current analysis were grains/potatoes, vegetables, and condiments. These foods are quite different from those in the typical diet of the Western population, in which a greater proportion of dietary iron is obtained from animal food [22], which indicated more heme iron intake. The dominance of plant-based food sources of dietary iron in the Chinese diet indicates a larger proportion of nonheme iron. In a Chinese study, no definite association was found for heme iron intake with iron status [25]. Another study on a Japanese population also found that heme iron intake is not correlated with iron status [30]. The difference between Western and Chinese dietary patterns could partially explain the difference in the associations of heme iron with MetS between the present findings and results from Western populations. Heme iron has been widely acknowledged to have better bioavailability than nonheme iron [31]. However, the ascorbic acid found in fruits and vegetables can enhance nonheme iron absorption [13]. In a study focusing on Chinese females, nonheme iron intake was found to be associated with serum ferritin [25]. This finding could support the correlation between nonheme iron intake and MetS observed in the present study.

In this study, higher risks of the four components of MetS, including elevated waist circumference, elevated triglycerides, reduced HDL-C, and elevated blood pressure, were observed with moderately higher total iron intake (the third quartile of total iron intake). Moderately higher nonheme iron intake (the third quartile of nonheme iron intake) was associated with higher risks of all five components of MetS. However, no associations were found between heme iron intake and the five components of MetS. Meanwhile, previous published studies indicated that iron overload in the body is correlated with all features of abnormal metabolism, such as obesity, hypertension, hyperlipidemia, and hyperglycemia [5,9,19,32,33].

We found that total iron and nonheme iron intake was associated with risk of MetS in male participants, but an association between iron intake and MetS was not observed in female participants in the current study. In the male population, the highest quartile of total iron intake was associated with a 2.1-fold higher risk of MetS compared with the lowest quartile. The Chinese recommended daily iron intakes for male and female adults are 12 mg and 20 mg, respectively, and the tolerable upper iron intake is 42 mg [34]. In this study, although the average dietary iron intake of both genders was far from the tolerable upper limit, the average total dietary iron intake of the male participants was almost double the recommended level, whereas that of the females was slightly under the recommended level. In addition, women experience greater iron loss than men due to menses. Increased iron stores are correlated with markers of chronic inflammation and risk of MetS [35,36]. The relatively higher iron intake and lower iron loss in men might explain the gender difference in the association between dietary iron intake and the occurrence of MetS observed in this study. Assessing the blood loss in the case of women warrants further study.

A limitation of this study is the methodology used to assess dietary intake. We used 3-day, 24-h dietary records to obtain food consumption information, and this information was then used to calculate the total, heme, and nonheme iron intake based on Chinese food composition data. Thus, the estimates of dietary iron intake were limited by the accuracy of the participants’ recall and estimation. Furthermore, although we adjusted for several potential confounding factors, including dietary energy intake, we did not treat other iron intake-related dietary components, such as saturated fat or zinc, as covariates to avoid multicollinearity. Therefore, we cannot avoid the possibility that recall bias, nonresponse bias, and other unknown confounding factors might influence the result of the risk factor analysis. Finally, it is logical to hypothesize that dietary iron intake influences the body’s metabolism, but the cross-sectional nature of the current study did not allow us to draw causal associations between total, heme, and nonheme dietary iron intake and MetS. Therefore, prospective observational studies or random clinical trials are needed to clarify the causal relationship between dietary iron and MetS.

## 5. Conclusions

Total and nonheme dietary iron intake was positively associated with MetS and its components in the adult population in metropolitan China, but an association between heme iron intake and MetS was not observed in this population. Moreover, a gender difference in the association between dietary iron intake and the occurrence of MetS was found: higher levels of total and nonheme iron intake were associated with greater risks of MetS in men but not in women.

## Figures and Tables

**Table 1:** Characteristics of the participants by gender in the SDHS 2012–2013.

	All	Male	Female	*p*-Value
*n* (%)	3099 (100.0)	1430 (46.1)	1669 (53.9)	
Age, %				0.513
18–44 years	30.8	30.1	31.4	
45–59 years	36.2	36.3	36.1	
60-years	33.0	33.6	32.6	
Yearly Income, %				0.523
Above average level (>60,000 RMB ^1^)	7.2	7.0	7.4	
Average level (30,000–59, RMB)	30.9	32.1	30.0	
Below average level (<30,000 RMB)	57.2	56.0	58.2	
No answer	4.7	4.9	4.5	
Years of Education, years (SD ^2^)	9.5 (4.53)	10.2 (4.01)	8.9 (4.86)	<0.001
Physical Activity Level, %				<0.001
Sedentary	84.2	78.7	89.0	
Moderate	13.8	18.1	10.2	
Vigorous	2.0	3.3	0.8	
Intentional Physical Exercise, %	24.8	25.2	24.5	0.940
Smoking Status, %				
Never smoked	72.0	40.8	98.8	
Former smoker	5.0	10.5	0.3	
Current smoker	23.0	48.8	1.0	
Alcohol Use, %				<0.001
Lifetime abstainers	80.7	64.0	94.9	
Nonheavy drinkers	15.1	27.3	4.6	
Infrequent heavy drinkers	1.3	2.6	0.3	
Frequent heavy drinkers	3.0	6.1	0.3	
Dietary Intake				
Energy, kcal/day (SD)	1760.9 (842.5)	1938.5 (884.5)	1608.9(773.4)	<0.001
Carbohydrate, g/day (SD)	207.4(119.3)	226.8(131.5)	190.8(105.1)	<0.001
Protein, g/day (SD)	67.9(45.6)	75.0(53.1)	61.9(37.1)	<0.001
Total fat, g/day (SD)	74.6(41.8)	81.1(40.7)	69.0(41.9)	<0.001
Total iron, mg/day (SD)	19.7(16.3)	22.0(20.4)	17.7(11.3)	<0.001
Heme iron, mg/day (SD)	1.6(1.4)	1.7(1.4)	1.5(1.4)	<0.001
Nonheme iron, mg/day (SD)	18.1(15.8)	20.3(20.0)	16.2(10.6)	<0.001
Metabolic Syndrome, %	23.9	21.8	25.7	0.011
Metabolic Syndrome Components				
Elevated blood pressure, %	52.4	56.8	48.7	<0.001
Elevated waist circumference, %	33.6	25.5	40.5	<0.001
Elevated fasting glucose, %	23.1	25.4	21.1	0.007
Elevated triglycerides, %	27.2	30.3	24.4	<0.001
Reduced HDL-C ^3^, %	21.0	14.8	26.2	<0.001

^1^ RMB, renminbi, China’s currency; ^2^ SD, standard deviation; ^3^ HDL-C, high-density lipoprotein-cholesterol.

**Table 2:** Odds ratios (ORs) (95% CI) for metabolic syndrome and its components according to the quartiles of total iron intake (mg/day) among all participants in the SDHS 2012–2013.

	Quartiles of Dietary Iron Intake, ORs (95% CI)				
	Q1	Q2	Q3	Q4	*p*-Value for Trend ^1^
**Total Iron Intake (mg/day)**	<12.72	(12.72–16.50)	(16.50–21.88)	≥21.88	
***n***	776	773	776	774	
**Metabolic syndrome**					
Crude model	Reference	1.12(0.88,1.42)	1.24(0.98,1.57)	1.17(0.92,1.48)	0.335
Adjusted model ^2^	Reference	1.37(1.06,1.78) *	1.47(1.11,1.94) **	1.59(1.15,2.20) **	0.022
				*p*-Value for interaction ^3^ = 0.060	
**Metabolic syndrome components**					
Elevated blood pressure					
Crude model	Reference	0.95(0.79,1.13)	1.19(1.00,1.42) *	1.07(0.89,1.27)	0.063
Adjusted model ^2^	Reference	1.14(0.92,1.41)	1.33(1.06,1.66) *	1.21(0.93,1.57)	0.100
Elevated waist circumference					
Crude model	Reference	1.07(0.89,1.29)	1.09(0.91,1.32)	1.00(0.83,1.21)	0.691
Adjusted model ^2^	Reference	1.25(1.01,1.53) *	1.29(1.04,1.60) *	1.25(0.96,1.61)	0.096
Elevated fasting glucose					
Crude model	Reference	1.03(0.83,1.27)	1.16(0.94,1.42)	1.12(0.91,1.38)	0.458
Adjusted model ^2^	Reference	1.18(0.94,1.49)	1.25(0.98,1.60)	1.33(1.00,1.77)	0.221
Elevated triglycerides					
Crude model	Reference	1.17(0.95,1.43)	1.46(1.20,1.77) **	1.34(1.10,1.64) **	0.001
Adjusted model ^2^	Reference	1.14(0.92,1.42)	1.37(1.09,1.72) **	1.31(1.00,1.71) *	0.047
Reduced HDL-C ^4^					
Crude model	Reference	1.09(0.86,1.38)	1.11(0.87,1.41)	0.91(0.71,1.16)	0.348
Adjusted model ^2^	Reference	1.27(0.98,1.65)	1.42(1.08,1.87) *	1.31(0.94,1.83)	0.088

* *p* < 0.05, ** *p* < 0.001. ^1^ The *p*-value for the trend was examined using the medians in each quartile of dietary iron intake. ^2^ The models were adjusted for age, sex, income, physical activity level, intentional physical exercise, smoking status, alcohol use and dietary total energy intake. ^3^ The *p*-value for the interaction was tested for the quartiles of dietary iron intake by gender. ^4^ HDL-C: high-density lipoprotein-cholesterol.

**Table 3:** ORs (95% CI) for metabolic syndrome and its components according to the quartiles of heme iron intake (mg/day) among all participants in the SDHS 2012–2013.

	Quartiles of Dietary Iron Intake, ORs (95% CI)				
	Q1	Q2	Q3	Q4	*p*-Value for Trend ^1^
**Heme Iron Intake (mg/day)**	<0.83	(0.83–1.28)	(1.28–1.94)	≥1.94	
***n***	776	774	773	776	
**Metabolic syndrome**					
Crude model	Reference	0.91(0.72,1.15)	0.92(0.73,1.16)	0.85(0.67,1.07)	0.593
Adjusted model ^2^	Reference	1.06(0.82,1.36)	1.11(0.86,1.44)	1.06(0.80,1.39)	0.895
				*p*-Value for interaction ^3^ = 0.096	
**Metabolic syndrome components**					
Elevated blood pressure					
Crude model	Reference	0.76(0.64,0.91) **	0.73(0.61,0.87) **	0.73(0.61,0.87) **	0.001
Adjusted model ^2^	Reference	0.91(0.74,1.13)	0.98(0.79,1.22)	1.05(0.83,1.32)	0.620
Elevated waist circumference					
Crude model	Reference	0.89(0.74,1.08)	0.95(0.79,1.14)	0.93(0.77,1.12)	0.681
Adjusted model ^2^	Reference	1.07(0.87,1.31)	1.20(0.98,1.48)	1.24(0.99,1.55)	0.189
Elevated fasting glucose					
Crude model	Reference	0.90(0.73,1.11)	0.82(0.67,1.01)	0.89(0.73,1.10)	0.330
Adjusted model ^2^	Reference	1.02(0.82,1.28)	0.96(0.76,1.21)	1.11(0.87,1.41)	0.661
Elevated triglycerides					
Crude model	Reference	1.05(0.86,1.29)	1.12(0.92,1.36)	1.08(0.89,1.32)	0.723
Adjusted model ^2^	Reference	1.03(0.83,1.28)	1.10(0.88,1.36)	1.00(0.79,1.26)	0.784
Reduced HDL-C ^4^					
Crude model	Reference	1.05(0.83,1.35)	1.16(0.91,1.47)	0.99(0.78,1.27)	0.547
Adjusted model ^2^	Reference	1.17(0.91,1.52)	1.29(0.99,1.68)	1.15(0.87,1.53)	0.291

* *p* < 0.05, ** *p* < 0.001. ^1^ The *p*-value for the trend was examined using the medians in each quartile of dietary iron intake. ^2^ The models were adjusted for age, sex, income, physical activity level, intentional physical exercise, smoking status, alcohol use and dietary total energy intake. ^3^ The *p*-value for the interaction was tested for the quartiles of dietary iron intake by gender. ^4^ HDL-C: high-density lipoprotein-cholesterol.

**Table 4:** ORs (95% CI) for metabolic syndrome and its components according to the quartiles of nonheme iron intake (mg/day) among all participants in the SDHS 2012–2013.

	Quartiles of Dietary Iron Intake, ORs (95% CI)				
	Q1	Q2	Q3	Q4	*p*-Value for Trend ^1^
**Nonheme Iron Intake (mg/day)**	<11.62	(11.62–15.10)	(15.10–20.10)	≥20.10	
***n***	774	774	775	776	
**Metabolic syndrome**					
Crude model	Reference	1.07(0.84,1.36)	1.23(0.97,1.55)	1.12(0.89,1.42)	0.364
Adjusted model ^2^	Reference	1.28(0.98,1.66)	1.43(1.09,1.87) *	1.44(1.04,1.99) *	0.065
				*p*-Value for interaction ^3^ = 0.086	
**Metabolic syndrome components**					
Elevated blood pressure					
Crude model	Reference	0.91(0.77,1.09)	1.26(1.06,1.50) **	1.08(0.91,1.29)	0.003
Adjusted model ^2^	Reference	1.07(0.86,1.32)	1.34(1.07,1.67) *	1.18(0.91,1.53)	0.058
Elevated waist circumference					
Crude model	Reference	1.07(0.89,1.29)	1.10(0.91,1.32)	1.00(0.83,1.20)	0.662
Adjusted model ^2^	Reference	1.22(0.99,1.50)	1.26(1.02,1.57) *	1.19(0.92,1.53)	0.149
Elevated fasting glucose					
Crude model	Reference	0.92(0.74,1.14)	1.19(0.97,1.46)	1.08(0.88,1.33)	0.088
Adjusted model ^2^	Reference	1.01(0.80,1.28)	1.28(1.01,1.63) *	1.25(0.94,1.66)	0.107
Elevated triglycerides					
Crude model	Reference	1.21(0.99,1.48)	1.42(1.17,1.73) **	1.34(1.10,1.64) **	0.003
Adjusted model ^2^	Reference	1.15(0.93,1.44)	1.33(1.06,1.66) *	1.26(0.96,1.64)	0.100
Reduced HDL-C ^4^					
Crude model	Reference	1.11(0.87,1.40)	1.06(0.83,1.35)	0.91(0.71,1.16)	0.425
Adjusted model ^2^	Reference	1.29(0.99,1.67)	1.34(1.02,1.76) *	1.30(0.93,1.81)	0.157

* *p* < 0.05, ** *p* < 0.001. ^1^ The *p*-value for the trend was examined using the medians in each quartile of dietary iron intake. ^2^ The models were adjusted for age, sex, income, physical activity level, intentional physical exercise, smoking status, alcohol use, and dietary total energy intake. ^3^ The *p*-value for the interaction was tested for the quartiles of dietary iron intake by gender. ^4^ HDL-C: high-density lipoprotein-cholesterol.

**Table 5:** ORs (95% CI) for metabolic syndrome and its components according to the quartiles of total iron intake (mg/day) among men in the SDHS 2012–2013.

	Quartiles of Dietary Iron Intake, ORs (95% CI)				
	Q1	Q2	Q3	Q4	*p*-Value for Trend ^1^
**Total Iron Intake (mg/day)**	<14.12	(14.12–17.87)	(17.87–23.68)	≥23.68	
***n***	357	358	358	357	
**Metabolic syndrome**					
Crude model	Reference	1.22(0.84,1.77)	1.66(1.16,2.39) **	1.65(1.15,2.36) **	0.013
Adjusted model ^2^	Reference	1.36(0.90,2.07)	1.83(1.19,2.81) **	2.11(1.29,3.45) **	0.016
**Metabolic syndrome components**					
Elevated blood pressure					
Crude model	Reference	1.07(0.83,1.38)	1.15(0.90,1.48)	1.16(0.90,1.49)	0.619
Adjusted model ^2^	Reference	1.35(1.00,1.84)	1.33(0.97,1.82)	1.43(0.99,2.06)	0.164
Elevated waist circumference					
Crude model	Reference	1.65(1.21,2.24) **	1.74(1.29,2.36) **	1.84(1.36,2.49) **	0.000
Adjusted model ^2^	Reference	1.59(1.14,2.21) **	1.61(1.15,2.27) **	1.65(1.12,2.43) *	0.019
Elevated fasting glucose					
Crude model	Reference	0.94(0.70,1.26)	1.00(0.75,1.33)	0.98(0.74,1.31)	0.973
Adjusted model ^2^	Reference	1.14(0.82,1.58)	1.17(0.83,1.66)	1.40(0.94,2.10)	0.431
Elevated triglycerides					
Crude model	Reference	1.24(0.94,1.64)	1.38(1.05,1.82) *	1.32(1.01,1.74) *	0.096
Adjusted model ^2^	Reference	1.21(0.89,1.65)	1.30(0.95,1.78)	1.34(0.93,1.94)	0.361
Reduced HDL-C					
Crude model	Reference	0.98(0.65,1.48)	1.23(0.83,1.83)	1.08(0.72,1.62)	0.658
Adjusted model ^2^	Reference	0.92(0.59,1.45)	1.38(0.87,2.19)	1.34(0.77,2.32)	0.287

* *p* < 0.05, ** *p* < 0.001. ^1^ The *p*-value for the trend was examined using the medians in each quartile of dietary iron intake. ^2^ The models were adjusted for age, income, physical activity level, intentional physical exercise, smoking status, alcohol use, and dietary total energy intake.

**Table 6:** ORs (95% CI) for metabolic syndrome and its components according to the quartiles of heme iron intake (mg/day) among men in the SDHS 2012–2013.

	Quartiles of Dietary Iron Intake, ORs (95% CI)				
	Q1	Q2	Q3	Q4	*p*-Value for Trend ^1^
**Heme Iron Intake (mg/day)**	<0.93	(0.93–1.40)	(1.40–2.02)	≥2.02	
***n***	357	358	357	358	
**Metabolic syndrome**					
Crude model	Reference	1.27(0.89,1.81)	1.09(0.76,1.56)	1.21(0.85,1.73)	0.547
Adjusted model ^2^	Reference	1.38(0.94,2.02)	1.12(0.75,1.68)	1.21(0.80,1.82)	0.415
**Metabolic syndrome components**					
Elevated blood pressure					
Crude model	Reference	0.99(0.76,1.28)	0.73(0.57,0.95) *	0.85(0.66,1.09)	0.059
Adjusted model ^2^	Reference	1.29(0.95,1.75)	1.05(0.77,1.43)	1.23(0.89,1.69)	0.308
Elevated waist circumference					
Crude model	Reference	1.25(0.91,1.69)	1.37(1.01,1.86) *	1.82(1.36,2.45) **	0.001
Adjusted model ^2^	Reference	1.26(0.91,1.76)	1.33(0.95,1.86)	1.89(1.35,2.65) **	0.003
Elevated fasting glucose					
Crude model	Reference	1.02(0.76,1.36)	0.85(0.63,1.14)	0.92(0.69,1.23)	0.597
Adjusted model ^2^	Reference	1.12(0.82,1.54)	1.02(0.73,1.42)	1.18(0.84,1.65)	0.729
Elevated triglycerides					
Crude model	Reference	1.02(0.78,1.35)	1.06(0.81,1.39)	1.01(0.77,1.33)	0.978
Adjusted model ^2^	Reference	0.90(0.67,1.22)	0.96(0.71,1.30)	0.82(0.59,1.12)	0.607
Reduced HDL-C					
Crude model	Reference	1.06(0.71,1.59)	1.03(0.69,1.54)	1.06(0.71,1.58)	0.991
Adjusted model ^2^	Reference	1.05(0.68,1.61)	0.96(0.62,1.49)	0.98(0.62,1.55)	0.982

* *p* < 0.05, ** *p* < 0.001. ^1^ The *p*-value for the trend was examined using the medians in each quartile of dietary iron intake. ^2^ The models were adjusted for age, income, physical activity level, intentional physical exercise, smoking status, alcohol use, and dietary total energy intake.

**Table 7:** ORs (95% CI) for metabolic syndrome and its components according to the quartiles of nonheme iron intake (mg/day) among men in the SDHS 2012–2013.

	Quartiles of Dietary Iron Intake, ORs (95% CI)				
	Q1	Q2	Q3	Q4	*p*-Value for Trend ^1^
**Nonheme Iron Intake (mg/day)**	<12.76	(12.76–16.31)	(16.31–21.72)	≥21.72	
***n***	358	357	357	358	
**Metabolic syndrome**					
Crude model	Reference	1.29(0.89,1.87)	1.65(1.15,2.37) **	1.60(1.11,2.30) *	0.026
Adjusted model ^2^	Reference	1.49(0.99,2.25)	1.79(1.17,2.74) **	2.05(1.26,3.35) **	0.025
**Metabolic syndrome components**					
Elevated blood pressure					
Crude model	Reference	0.97(0.75,1.25)	1.16(0.91,1.50)	1.11(0.86,1.43)	0.443
Adjusted model ^2^	Reference	1.21(0.89,1.64)	1.24(0.91,1.70)	1.28(0.88,1.84)	0.494
Elevated waist circumference					
Crude model	Reference	1.43(1.06,1.94) *	1.49(1.10,2.01) **	1.71(1.27,2.31) **	0.004
Adjusted model ^2^	Reference	1.40(1.01,1.94) *	1.37(0.98,1.92)	1.48(1.01,2.17) *	0.143
Elevated fasting glucose					
Crude model	Reference	0.86(0.64,1.15)	0.94(0.71,1.25)	0.97(0.72,1.29)	0.769
Adjusted model ^2^	Reference	1.02(0.74,1.42)	1.10(0.78,1.55)	1.33(0.89,1.98)	0.500
Elevated triglycerides					
Crude model	Reference	1.28(0.97,1.69)	1.37(1.04,1.79) *	1.31(0.99,1.73)	0.113
Adjusted model ^2^	Reference	1.25(0.92,1.69)	1.23(0.90,1.69)	1.33(0.92,1.92)	0.399
Reduced HDL-C					
Crude model	Reference	1.04(0.70,1.57)	1.20(0.81,1.78)	1.06(0.71,1.58)	0.822
Adjusted model ^2^	Reference	1.06(0.68,1.65)	1.38(0.87,2.20)	1.35(0.78,2.33)	0.495

* *p* < 0.05, ** *p* < 0.001. ^1^ The *p*-value for the trend was examined using the medians in each quartile of dietary iron intake. ^2^ The models were adjusted for age, income, physical activity level, intentional physical exercise, smoking status, alcohol use, and dietary total energy intake.

**Table 8:** ORs (95% CI) for metabolic syndrome and its components according to the quartiles of total iron intake (mg/day) among women in the SDHS 2012–2013.

	Quartiles of Dietary Iron Intake, ORs (95% CI)				
	Q1	Q2	Q3	Q4	*p*-Value for Trend ^1^
**Total Iron Intake (mg/day)**	<11.67	(11.67–15.10)	(15.10–20.00)	≥20.00	
***n***	416	419	416	418	
**Metabolic syndrome**					
Crude model	Reference	1.02(0.74,1.40)	1.21(0.88,1.65)	0.96(0.70,1.32)	0.476
Adjusted model ^2^	Reference	1.25(0.88,1.78)	1.41(0.98,2.02)	1.27(0.82,1.96)	0.322
**Metabolic syndrome components**					
Elevated blood pressure					
Crude model	Reference	0.86(0.68,1.10)	1.08(0.85,1.37)	0.85(0.67,1.09)	0.163
Adjusted model ^2^	Reference	1.01(0.75,1.37)	1.29(0.94,1.76)	1.05(0.73,1.52)	0.306
Elevated waist circumference					
Crude model	Reference	0.97(0.76,1.24)	1.01(0.79,1.29)	0.93(0.72,1.19)	0.914
Adjusted model ^2^	Reference	1.09(0.83,1.44)	1.13(0.85,1.50)	1.15(0.82,1.62)	0.839
Elevated fasting glucose					
Crude model	Reference	0.90(0.67,1.22)	1.23(0.92,1.64)	1.09(0.81,1.47)	0.216
Adjusted model ^2^	Reference	1.01(0.73,1.41)	1.33(0.95,1.85)	1.23(0.83,1.83)	0.274
Elevated triglycerides					
Crude model	Reference	1.01(0.75,1.34)	1.13(0.85,1.50)	1.08(0.81,1.44)	0.814
Adjusted model ^2^	Reference	1.06(0.77,1.44)	1.16(0.85,1.60)	1.18(0.81,1.72)	0.773
Reduced HDL-C					
Crude model	Reference	1.19(0.88,1.62)	1.14(0.84,1.56)	1.14(0.84,1.56)	0.710
Adjusted model ^2^	Reference	1.37(0.98,1.91)	1.31(0.93,1.85)	1.40(0.93,2.10)	0.264

^1^ The *p*-value for the trend was examined using medians in each quartile of dietary iron intake. ^2^ The models were adjusted for age, income, physical activity level, intentional physical exercise, smoking status, alcohol use, and dietary total energy intake.

**Table 9:** ORs (95% CI) for metabolic syndrome and its components according to the quartiles of heme iron intake (mg/day) among women in the SDHS 2012–2013.

	Quartiles of Dietary Iron Intake, ORs (95% CI)				
	Q1	Q2	Q3	Q4	*p*-Value for Trend ^1^
**Heme Iron Intake (mg/day)**	<0.76	(0.76–1.19)	(1.19–1.81)	≥1.81	
***n***	416	419	419	415	
**Metabolic syndrome**					
Crude model	Reference	0.88(0.65,1.19)	0.81(0.60,1.11)	0.74(0.54,1.01)	0.273
Adjusted model ^2^	Reference	1.08(0.78,1.52)	1.06(0.75,1.51)	1.08(0.74,1.57)	0.967
**Metabolic syndrome components**					
Elevated blood pressure					
Crude model	Reference	0.71(0.55,0.90) **	0.63(0.49,0.80) **	0.58(0.45,0.74) **	0.000
Adjusted model ^2^	Reference	0.93(0.69,1.26)	0.90(0.67,1.23)	0.91(0.66,1.26)	0.924
Elevated waist circumference					
Crude model	Reference	0.94(0.74,1.21)	0.91(0.71,1.16)	0.77(0.59,0.99) *	0.193
Adjusted model ^2^	Reference	1.18(0.90,1.54)	1.22(0.92,1.61)	1.05(0.78,1.42)	0.439
Elevated fasting glucose					
Crude model	Reference	0.85(0.63,1.13)	0.75(0.55,1.00)	0.87(0.65,1.17)	0.285
Adjusted model ^2^	Reference	0.98(0.72,1.35)	0.94(0.67,1.30)	1.12(0.79,1.58)	0.755
Elevated triglycerides					
Crude model	Reference	1.06(0.79,1.41)	1.01(0.76,1.35)	1.00(0.75,1.34)	0.979
Adjusted model ^2^	Reference	1.17(0.87,1.59)	1.14(0.83,1.57)	1.20(0.86,1.67)	0.700
Reduced HDL-C					
Crude model	Reference	1.13(0.83,1.55)	1.54(1.13,2.09) **	1.11(0.81,1.53)	0.033
Adjusted model ^2^	Reference	1.24(0.89,1.73)	1.65(1.18,2.31) **	1.25(0.87,1.79)	0.027

* *p* < 0.05, ** *p* < 0.001. ^1^ The *p*-value for the trend was examined using medians in each quartile of dietary iron intake. ^2^ The models were adjusted for age, income, physical activity level, intentional physical exercise, smoking status, alcohol use, and dietary total energy intake.

**Table 10:** ORs (95% CI) for metabolic syndrome and its components according to the quartiles of nonheme iron intake (mg/day) among women in the SDHS 2012–2013.

	Quartiles of Dietary Iron Intake, ORs (95% CI)				
	Q1	Q2	Q3	Q4	*p*-Value for Trend ^1^
**Nonheme Iron Intake (mg/day)**	<10.69	(10.69–13.76)	(13.76–18.24)	≥18.24	
***n***	418	418	416	417	
**Metabolic syndrome**					
Crude model	Reference	0.87(0.64,1.21)	1.25(0.92,1.70)	0.92(0.67,1.27)	0.111
Adjusted model ^2^	Reference	1.01(0.71,1.44)	1.42(0.99,2.03)	1.14(0.74,1.75)	0.150
**Metabolic syndrome components**					
Elevated blood pressure					
Crude model	Reference	0.93(0.73,1.18)	1.17(0.92,1.49)	0.93(0.73,1.19)	0.214
Adjusted model ^2^	Reference	1.10(0.81,1.49)	1.42(1.03,1.94) *	1.15(0.80,1.66)	0.140
Elevated waist circumference					
Crude model	Reference	0.90(0.70,1.15)	1.00(0.78,1.28) **	0.90(0.70,1.15)	0.689
Adjusted model ^2^	Reference	0.99(0.75,1.30)	1.08(0.81,1.44)	1.05(0.74,1.47)	0.918
Elevated fasting glucose					
Crude model	Reference	0.96(0.71,1.31)	1.29(0.97,1.73)	1.16(0.86,1.56)	0.181
Adjusted model ^2^	Reference	1.09(0.78,1.51)	1.40(1.00,1.95) *	1.29(0.87,1.91)	0.215
Elevated triglycerides					
Crude model	Reference	0.92(0.68,1.23)	1.24(0.94,1.64)	1.02(0.76,1.35)	0.194
Adjusted model ^2^	Reference	0.94(0.68,1.28)	1.26(0.92,1.72)	1.06(0.73,1.54)	0.243
Reduced HDL-C					
Crude model	Reference	1.19(0.87,1.62)	1.16(0.85,1.58)	1.07(0.78,1.47)	0.685
Adjusted model ^2^	Reference	1.30(0.93,1.81)	1.28(0.91,1.80)	1.23(0.82,1.85)	0.421

* *p* < 0.05, ** *p* < 0.001. ^1^ The *p*-value for the trend was examined using medians in each quartile of dietary iron intake. ^2^ The models were adjusted for age, income, physical activity level, intentional physical exercise, smoking status, alcohol use, and dietary total energy intake.

## Appendix A

**Table A1:** ORs (95% CI) for metabolic syndrome according to the quartiles of total iron intake (mg/day) by age group in the SDHS 2012–2013.

	Quartiles of Dietary Iron Intake, ORs (95% CI)				
	Q1	Q2	Q3	Q4	*p*-Value for Trend ^1^
**18–44 years**					
**Total Iron Intake (mg/day)**	<12.84	(12.84–16.02)	(16.02–21.50)	≥21.50	
***n***	241	241	241	241	
**Metabolic syndrome**					
Crude model	Reference	1.55(0.81,2.94)	1.72(0.92,3.24)	1.88(1.00,3.51) *	0.237
Adjusted model ^2^	Reference	1.53(0.77,3.03)	1.66(0.82,3.34)	1.96(0.86,4.46)	0.430
**45–59 years**					
**Total Iron Intake (mg/day)**	<13.03	(13.03–17.04)	(17.04–20.00)	≥22.31	
***n***	280	279	279	279	
**Metabolic syndrome**					
Crude model	Reference	1.10(0.74,1.65)	1.35(0.91,2.00)	1.05(0.70,1.58)	0.448
Adjusted model ^2^	Reference	1.16(0.77,1.76)	1.41(0.92,2.16)	1.18(0.71,1.96)	0.458
**60-years**					
**Total Iron Intake (mg/day)**	<12.31	(12.31–16.28)	(16.28–20.00)	≥21.86	
***n***	255	254	255	254	
**Metabolic syndrome**					
Crude model	Reference	1.03(0.70,1.50)	1.11(0.77,1.62)	1.03(0.70,1.51)	0.949
Adjusted model ^2^	Reference	1.24(0.83,1.86)	1.54(1.00,2.37) *	1.77(1.06,2.96) *	0.135

* *p* < 0.05. ^1^ The *p*-value for the trend was examined using the medians in each quartile of dietary iron intake. ^2^ The models were adjusted for sex, income, physical activity level, intentional physical exercise, smoking status, alcohol use, and dietary total energy intake.
